# The impact of sociodemographic factors on the utilization of radiation therapy in breast cancer patients in Estonia: a register-based study

**DOI:** 10.1186/s12939-021-01497-0

**Published:** 2021-06-30

**Authors:** Fereshteh Shahrabi Farahani, Keiu Paapsi, Kaire Innos

**Affiliations:** 1 School of Information Technologies, Department of Health Technologies, Tallinn University of Technology, Digital Health MSc Programme, Tallinn, Estonia; 2 Department of Epidemiology and Biostatistics, National Institute for Health Development, Hiiu 42, 11619 Tallinn, Estonia

**Keywords:** Breast cancer, Radiation therapy, Stage, Social determinants, Education, Marital status, Estonia

## Abstract

**Background:**

Radiation therapy is an important part of multimodal breast cancer treatment. The aim was to examine the impact of sociodemographic factors on radiation therapy use in breast cancer (BC) patients in Estonia, linking cancer registry data to administrative databases.

**Methods:**

Estonian Cancer Registry provided data on women diagnosed with BC in Estonia in 2007–2018, including TNM stage at diagnosis. Use of radiation therapy within 12 months of diagnosis was determined from Estonian Health Insurance Funds claims, and sociodemographic characteristics from population registry. Receipt of radiation therapy was evaluated over time and by clinical and sociodemographic factors. Poisson regression with robust variance was used to calculate univariate and multivariate prevalence rate ratios (PRR) with 95 % confidence intervals (CI) for receipt of radiation therapy among stage I–III BC patients age < 70 years who underwent primary surgery.

**Results:**

Overall, of 8637 women included in the study, 4310 (50 %) received radiation therapy within 12 months of diagnosis. This proportion increased from 39 to 58 % from 2007 to 2009 to 2016–2018 (*p* < 0.001). Multivariate regression analysis showed that compared to women with stage I BC, those with more advanced stage were less likely to receive radiation therapy. Receipt of radiation therapy increased significantly over time and was nearly 40 % higher in 2016–2018 than in 2007–2009. Use of radiation therapy was significantly lower for women with the lowest level of education compared to those with a university degree (PRR 0.88, 95 % CI 0.80–0.97), and for divorced/widowed women (PRR 0.95, 95 % CI 0.91–0.99) and single women (PRR 0.92, 95 % CI 0.86–0.99), compared to married women. Age at diagnosis, nationality and place of residence were not associated with receipt of radiation therapy.

**Conclusions:**

The study showed considerable increase in the use of radiation therapy in Estonia over the study period, which is in line with increases in available equipment. The lack of geographic variations suggests equal access to therapy for patients living in remote regions. However, educational level and marital status were significantly associated with receipt of radiation therapy, highlighting the importance of psychosocial support in ensuring equal access to care.

## Background

Breast cancer (BC) is a major health burden among women worldwide as well as in Estonia. Although overall BC incidence has increased in Estonia [1], mortality from BC has decreased steadily since 2000 [2]. However, there’s still a survival gap between Estonia and more developed countries [3], despite rapid increase observed since 1990s, particularly for locally/regionally spread cancers [4]. BC five-year relative survival ratio in Estonia was 81 % in 2012–2016 [5], whereas the survival of BC patients in the Nordic countries and England was approximately 90 and 85 %, respectively, for the same period [6, 7].

The most likely reason for inferior survival is late diagnosis, as the proportion of BC cases diagnosed at early stage is lower in Estonia than in many other European countries [8]. However, the role of treatment should be considered as well.

Radiation therapy (RT) is an essential part of multimodal BC treatment. In early BC, RT is the standard of care after breast-conserving surgery (BCS) and is sometimes also indicated after mastectomy [9]. RT helps to decrease the risk of local recurrence or death in BC patients after BCS [10–12]. Patients with locally advanced disease may receive RT in combination with surgery and systemic therapy, and those with metastatic disease, to relieve symptoms and improve quality of life [13].

The number of RT equipment in Estonia was one of the lowest in Europe in 2012, with 3.0 megavoltage (MV) units per million population compared to the median of 5.3 of 28 European countries [14]. RT in Estonia is done at two specialist cancer centres, located in Tallinn (the capital of Estonia) and in Tartu (a university town in Southern Estonia). During 2007–2011, three MV units were in use; one was added in 2011, and two more in 2016, which currently totals 4.6 MV units per million population [14, 15].

Estonia has a population of 1.3 million, two-thirds of which are ethnic Estonians and one-fourth are Russians. After regaining independence in 1991 and the stabilization of the political situation, fundamental health care reforms took place, including mandatory health insurance and restructured hospital network. More recent adjustments have been marginal, aiming to harmonize the framework with EU legislation. Health insurance tax is paid by employers for their employees, and by the state for other categories of insured people (e.g., children, retired and unemployed persons, pregnant women). Health insurance covers a broad range of curative and preventive services, including standard cancer care, based on contractual relations between Estonian Health Insurance Fund (EHIF) and service providers. In general, 95 % of the population is covered by health insurance [16].

Nevertheless, disparities in overall health status, cancer mortality, as well as BC early detection have been reported by educational level, nationality, or region of residence [17–20]. In particular, the North-Eastern industrial region bordering Russia has been previously recognized as problematic in terms of health [20]. The islands of Western Estonia and the North-Eastern region can be considered the most remote from cancer centres.

Internationally, social and geographic variations in receipt of BC treatments, including RT, have been widely documented in different settings and populations, revealing the unfavourable impact of geographic distance from services as well as barriers related to race/ethnicity or educational level [21–32].

The use of RT in women with BC in Estonia has previously been studied only within the framework of international high-resolution studies, demonstrating a drastic increase in the use of BCS + RT in early BC from 9 % to 1997 to 75 % in 2011 [8, 33]. Nevertheless, among nine European countries, the use of BCS + RT in Estonia was the second lowest [8]. There have been no studies examining receipt of RT in relation to individual sociodemographic factors in Estonia.

This study was designed within the framework of social determinants of cancer examining social gradients on health-care delivery level, trying to shed light on the effect of social and behavioural/psychological factors on access to and use of health services, linking data available from different registration systems [34].

Cancer cases were identified from the Estonian Cancer Registry (ECR), a population-based registry with nationwide coverage, which has reliable cancer incidence data from 1968 [5]. It is compulsory for all physicians and pathologists working in Estonia to report cancer cases to ECR. Additionally, ECR uses multiple sources to ascertain cancer cases including regular linkages with two cancer centres and trace-back of cases identified via death certificates. The completeness of case reporting is high as evidenced by data quality indicators [5]. Registry data on surgical treatment is considered rather reliable [35], but the completeness of RT data has not been evaluated. Failure of cancer registries to capture complete and accurate information on nonsurgical cancer treatment has been reported elsewhere [36, 37].

Therefore, RT data were collected from the central electronic database of EHIF which is a reimbursement database containing claims for all medical procedures performed in insured persons. Sociodemographic data were obtained from the population registry, a national database maintained and developed by Estonian government containing main personal information on all Estonian citizens and residents.

The aim of the study was to examine the utilization of RT among BC patients in Estonia over time, by sociodemographic factors and stage at diagnosis, combining data from the cancer registry and other population-based databases. An additional aim was to assess the completeness of RT data at the ECR.

## Materials and methods

### Data collection and definitions

Information on BC cases diagnosed in 2007–2018 was retrieved from the ECR. ECR uses the 3rd edition of International Classification of Diseases for Oncology (ICD-O-3) for coding topography and morphology of the tumours. ECR provided data on all invasive BC cases (ICD-O-3 topography codes C50.0–C50.9) diagnosed in Estonia between 2007 and 2018, regardless of cancer sequence (*n* = 8804). Data were collected using the same notification form during the study period. Male patients (*n* = 68), those with stage 0 disease (*n* = 10), death certificate only cases (*n* = 69), and autopsy cases (*n* = 20) were excluded from the analysis. The data obtained from the ECR included personal data, and data on diagnosis (date of diagnosis, age, and stage at diagnosis) and treatment (surgery and RT). Age at diagnosis was collapsed into four categories: <50, 50–59, 60–69, ≥ 70 years. Stage at diagnosis was categorized according to the Union for International Cancer Control TNM classification version 7. Period of diagnosis was divided into four three-year categories to account for the changes in the availability of RT equipment: 2007–2009, 2010–2012, 2013–2015 and 2016–2018. Region of residence was collapsed into five categories: Northern, Western, Central, North-Eastern and Southern Estonia. RT and surgery data included dates of treatment and description of procedures in text format.

Additional data on treatment was retrieved from the EHIF database which contains claims for all medical procedures performed in insured persons including dates of services and diagnostic codes according to International Statistical Classification of Diseases and Health Problems 10th Revision (ICD-10).

For BC cases included in the study, EHIF provided data on claims for RT (separately for RT planning and procedures), chemotherapy, and surgery for 2007–2019. The primary outcome was defined as receipt of RT, based on claims filed for at least one RT procedure performed within 12 months of diagnosis. The time frame was set to account for radiation performed during initial course of therapy. Time between diagnosis and RT was calculated from the date of cancer diagnosis to the date of starting RT. RT was considered as not received for cases for whom EHIF database included claims only for RT planning.

All patients with surgery reported within 12 months of diagnosis were considered as having had primary surgery. Type of surgery was categorised into BCS or mastectomy/other based on either procedure codes on insurance claims or description of surgery at ECR.

Data on sociodemographic variables for BC cases included in the study were obtained from the population registry. Nationality was grouped into Estonian, other nationalities, and unknown. Educational level was categorised as university and higher education, secondary studies plus vocational education, secondary studies, basic or primary studies and unknown. Marital status was classified as married, divorced/widowed, single, and unknown.

Data linkages were done using unique personal identification numbers, which have been in use in Estonia since 1992.

### RT data validation

The validity of EHIF RT data was checked against an existing high-resolution database collected for a previous study including diagnosis and treatment data for BC cases diagnosed in 2011 from the medical records of cancer centres and other hospitals [15].

As an additional analysis, we evaluated the completeness of RT data at the ECR, comparing RT data reported to the ECR on cancer notification form to RT data obtained from EHIF.

### Statistical analysis

Statistical analysis was performed with statistical software Stata 16 [38]. Chi-square test was used to compare proportions between groups. Two-sided *p*-value < 0.05 was considered statistically significant. The prevalence rate ratio (PRR) for receipt of RT with 95 % confidence intervals (CI) was calculated using univariate and multivariate Poisson regression models with robust variance, performed with generalized linear models with Poisson family and log link function in Stata. This method was selected because the odds ratio calculated with logistic regression tends to overestimate the association between variables when the prevalence is moderate to high [39]. In regression modelling, we included women with stage I–III cancer, age < 70 at diagnosis who underwent primary surgery, to account for treatment guidelines. Cases with ‘unknown’ educational level, marital status or nationality were also excluded from modelling. A sensitivity analysis was conducted with BCS + RT as an outcome variable among women with stage I–II cancer.

### Ethics

The study protocol was approved by the Tallinn Medical Research Ethics Committee.

## Results

### Patient characteristics

In total, 8637 women met the inclusion criteria for this study; the proportion of cases with microscopic verification was 97 % (Table 1). Overall, 49.9 % of the patients received RT. Receipt of RT increased considerably over time from 39.1 % in 2007–2009 to 57.9 % in 2016–2018), was highest in age group 50–59 (65.1 %, *p* < 0.001) and at stage I (66.1 %, *p* < 0.001). There was no difference in RT use by nationality. RT utilization was the highest in Southern Estonia (53.9 %) and the lowest in Western Estonia (45.3 %, *p* < 0.001). RT was used in 58.0 % of women with higher than secondary education compared to 28.8 % among women who had completed basic and primary studies (*p* < 0.001). The proportion of married women who received RT was 59.9 %, which was higher in comparison to single (50.0 %) and divorced/widowed women (43.2 %, *p* < 0.001 for both). Patients who received primary surgical treatment or chemotherapy, were more likely to receive RT than those who did not receive these treatments (*p* < 0.001 for both).

**Table 1:** Characteristics of women with breast cancer and receipt of radiation therapy, Estonia 2007–2018

			Receipt of radiation therapy				
			Yes		No		
Variable	No.	%	No.	row%	No.	row%	*p*-value^a^
Total	8637	100	4310	49.9	4327	50.1	
Microscopically verified	8387	97.1	4305	51.3	4082	48.7	<0.001
Period of diagnosis							
2007–2009	1939	22.5	758	39.1	1181	60.9	<0.001
2010–2012	2144	24.8	1041	48.6	1103	51.5	
2013–2015	2218	25.7	1158	52.2	1060	47.8	
2016–2018	2336	27.1	1353	57.9	983	42.1	
Age at diagnosis (years)							
<50	1523	17.6	943	61.9	580	38.1	<0.001
50–59	1993	23.1	1297	65.1	696	34.9	
60–69	2057	23.8	1230	59.8	827	40.2	
≥70	3064	35.5	840	27.4	2224	72.6	
TNM stage							
I	2483	28.8	1642	66.1	841	33.9	<0.001
II	3238	37.5	1531	47.3	1707	52.7	
III	1574	18.2	839	53.3	735	46.7	
IV	718	8.3	82	11.4	636	88.6	
Unknown	624	7.2	216	34.6	408	65.4	
Nationality							
Estonian	5490	63.6	2753	50.2	2737	49.9	0.163
Other nationalities	3112	36.0	1545	49.7	1567	50.4	
Unknown	35	0.4	12	34.3	23	65.7	
Region of residence							
North	4010	46.4	1991	49.7	2019	50.4	<0.001
West	927	10.7	420	45.3	507	54.7	
Central	704	8.2	347	49.3	357	50.7	
North-East	1077	12.5	518	48.1	559	51.9	
South	1919	22.2	1034	53.9	885	46.1	
Educational level							
University degree	1964	22.7	1139	58.0	825	42.0	<0.001
Secondary plus vocational studies	1860	21.5	1079	58.0	781	42.0	
Secondary studies	2593	30.0	1345	51.8	1248	48.1	
Basic and primary studies	1410	16.3	406	28.8	1004	71.2	
Unknown	810	9.4	341	42.1	469	57.9	
Marital Status							
Married	3035	35.1	1818	59.9	1217	40.1	<0.001
Divorced/widow	4389	50.8	1898	43.2	2491	56.8	
Single	976	11.3	488	50.0	488	50.0	
Unknown	237	2.7	106	44.7	131	55.3	
Primary surgical treatment							
Yes	7075	81.9	4230	59.8	2845	40.2	<0.001
No	1562	18.1	80	5.1	1482	94.9	
Chemotherapy							
Yes	5518	63.9	3147	57.0	2371	43.0	<0.001
No	3119	36.1	1163	37.3	1965	62.7	

Due to rounding, percentages may not total 100

^a^Chi-square test

Among women with stage I–III cancer aged < 70 years at diagnosis who underwent primary surgery, a significant increase in RT use was seen from 56.9 % in 2007–2009 to 78.3 % in 2016–2018 (Table 2). Significant increases over the study period were observed in all subgroups. The largest increase by stage was seen for stage III, from 54.6 to 86.1 %. RT utilization increased for all age groups, but the most among women aged 60–69, from 51.2 to 78.7 %. Among women with basic and primary educational level, receipt of RT showed a significant upward trend from 42.4 to 73.3 %; among divorced/widowed women, from 54.1 to 77.4 %.

**Table 2:** Receipt of radiation therapy over time, Estonia 2007–2018

	Receipt of radiation therapy									
	2007–2009 *N*=1116^a^		2010–2012 *N*=1185^a^		2013–2015 *N*=1153^a^		2016–2018 *N*=1248^a^			
	No.	row%	No.	row%	No.	row%	No.	row%	Change^b^	*p*-value^c^
Total	635	56.9	786	66.3	823	71.4	977	78.3	21.4	<0.001
Age at diagnosis (years)										
<50	178	58.0	222	67.1	191	62.8	263	77.1	19.1	<0.001
50–59	264	61.1	296	67.7	327	76.9	329	78.7	17.6	<0.001
60–69	193	51.2	268	64.3	305	71.9	385	78.7	27.5	<0.001
TNM stage										
I	266	72.7	309	75.6	370	80.8	407	80.1	7.4	0.014
II	219	46.1	314	57.3	303	61.3	390	73.5	27.4	<0.001
III	150	54.6	163	71.5	150	74.6	180	86.1	31.5	<0.001
Nationality^d^										
Estonian	403	57.2	493	68.6	502	69.6	608	78.7	21.5	<0.001
Other nationalities	230	56.2	292	63.1	320	74.4	366	77.5	21.3	<0.001
Region of residence										
North	272	55.1	371	66.5	405	71.6	466	76.4	21.3	<0.001
West	66	53.7	82	65.6	70	69.3	92	75.4	21.7	0.003
Central	54	55.1	58	66.7	62	66.0	92	79.3	24.2	0.002
North-East	85	59.0	100	62.5	98	72.6	121	82.9	23.9	<0.001
South	158	61.5	175	68.6	188	73.2	206	81.1	19.6	<0.001
Educational level^d^										
University degree	157	59.3	212	66.5	251	72.1	285	76.0	16.7	<0.001
Secondary plus vocational studies	162	59.8	211	68.3	217	73.3	266	78.7	18.9	<0.001
Secondary studies	222	56.6	258	64.7	257	72.8	316	80.2	23.6	<0.001
Basic and primary studies	42	42.4	52	64.2	53	58.2	55	73.3	30.9	<0.001
Marital status^d^										
Married	279	59.9	392	70.0	366	73.9	464	79.7	19.8	<0.001
Divorced/widow	272	54.1	289	63.5	337	70.7	352	77.4	23.3	<0.001
Single	67	55.8	92	63.0	92	64.8	127	74.3	18.5	0.011

^a^Includes women with stage I–III breast cancer, age <70 years who underwent primary surgery

^b^From first to last period

^c^Chi-square test

^d^Unknown category not shown

### Regression analysis

Table 3 presents the results of regression modelling for women with stage I–III cancer aged < 70 years at diagnosis who underwent primary surgery (*n* = 4312). In univariate analysis, receipt of RT was associated with period of diagnosis, age, stage, educational level, and marital status. After adjusting for other variables in multivariate analysis, a nearly 40 % increase in receipt of RT was observed over the study period (PRR 1.37, 95 % CI 1.29–1.45). Age showed a slight reverse U-shape association with RT use, which was borderline significant. RT use varied by stage, with stage I patients having the highest rates and stage II patients the lowest (PRR 0.79, 95 % CI 0.75–0.82). Women with the lowest level of education were significantly less likely to receive RT than other educational categories (PRR 0.88, 95 % CI 0.80–0.97). Also, compared to married women, lower rates of RT utilization were observed for divorced/widowed women (PRR 0.95, 95 % CI 0.91–0.99) and for single women (PRR 0.92, 95 % CI 0.86–0.99). No associations were observed in multivariate analysis across regions of residence or by nationality.

**Table 3:** Prevalence rate ratio of radiation therapy use among women with breast cancer, Estonia 2007–2018

	Radiation therapy use (*n*=4312) ^a^		Univariate PRR (95% CI)	Multivariate PRR (95% CI)
	No.	%		
Total	2954	68.5		
Period of diagnosis				
2007–2009	569	56.6	Ref	Ref
2010–2012	727	66.5	**1.18 (1.10–1.26)**	**1.18 (1.10–1.26)**
2013–2015	759	71.6	**1.27 (1.18–1.35)**	**1.26 (1.18–1.35)**
2016–2018	899	78.0	**1.38 (1.30–1.47)**	**1.37 (1.29–1.45)**
Age at diagnosis (years)				
<50	802	66.5	**0.94 (0.89–0.99)**	0.96 (0.91–1.01)
50–59	1126	70.9	Ref	Ref
60–69	1026	67.6	0.95 (0.91–1.00)	0.95 (0.91–1.00)
TNM stage				
I	1236	77.7	Ref	Ref
II	1140	60.2	**0.77 (0.74–0.81)**	**0.79 (0.75–0.82)**
III	578	69.9	**0.90 (0.85–0.95)**	**0.93 (0.88–0.98)**
Nationality				
Estonian	1805	68.9	Ref	Ref
Other nationalities	1149	67.9	0.98 (0.94–1.03)	0.98 (0.93–1.02)
Region of residence				
North	1340	67.6	Ref	Ref
West	296	65.8	0.97 (0.90–1.05)	0.99 (0.92–1.07)
Central	249	68.4	1.01 (0.94–1.09)	1.01 (0.94–1.09)
North-East	397	69.0	1.02 (0.96–1.09)	1.04 (0.98–1.11)
South	672	71.3	1.05 (1.00–1.11)	1.04 (0.99–1.10)
Educational level				
University degree	874	69.2	Ref	Ref
Secondary plus vocational studies	846	70.6	1.02 (0.97–1.07)	1.02 (0.97–1.08)
Secondary studies	1036	68.5	0.99 (0.94–1.04)	1.01 (0.96–1.06)
Basic and primary studies	198	58.6	**0.85 (0.77–0.93)**	**0.88 (0.80–0.97)**
Marital status				
Married	1395	71.2	Ref	Ref
Divorced/widowed	1193	66.6	**0.93 (0.90–0.98)**	**0.95 (0.91–0.99)**
Single	366	65.2	**0.92 (0.86–0.98)**	**0.92 (0.86–0.99**)

Statistically significant results in bold

Abbreviations: *PRR* prevalence rate ratio, *ref* reference category

^a^Includes women with stage I–III breast cancer, age <70 years who underwent primary surgery and had data on sociodemographic variables

In sensitivity analysis including only women with stage I–II cancer aged < 70 years at diagnosis who underwent primary surgery (*n* = 3485) and using BCS + RT as outcome variable, the PRRs remained similar, but confidence intervals widened.

### RT data validation

The primary outcome variable, receipt of RT within 12 months of diagnosis, identified from insurance claims, was validated against data collected for a previous high-resolution study. There was a 99.4 % agreement as two cases who were reported to have undergone RT according to medical records had no claims in the EHIF database.

Evaluation of the completeness of RT information at ECR showed that RT information was missing from the cancer registry for 31.2 % of cases for whom EHIF had claims for RT performed within 12 months of diagnosis. The proportion of RT information missing from ECR was 33 %, 27 %, 33 %, 28 and 38 % for stages I, II, III, IV and unknown, respectively. In addition, for 6.8 % of cases with reported RT, there was no date at ECR, and the timing of RT could not be assessed. The agreement for not receiving RT was 99.2 % between EHIF and ECR. Among the 33 cases with discordant information, 11 cases did not have any claims at EHIF, for 5 cases, there was a claim only for RT planning, and for 17 cases, RT was performed later that 12 months since diagnosis, but there was no date at ECR to assess the timing of RT.

## Discussion

In this population-based record linkage study of over 8600 women with BC, we found a significant impact of social factors such as the patients’ educational level and marital status on receipt of RT. We did not find significant associations between RT utilization and nationality or place of residence. The utilization of RT increased considerably over time, particularly for age group 60–69, stage III, and women with the lowest level of education.

The main strength of the study was the identification of BC cases from a high-quality cancer registry, with additional data on cancer treatment and sociodemographic factors obtained from two large national databases through individual linkages. Insurance claims have not been previously used to identify cancer treatment in Estonia. The validation of the primary RT variable against data collected from medical records showed a 99.4 % agreement between these databases. The likely explanation for the minor discrepancy (two cases with missing insurance claims) is that data from medical records were collected from multidisciplinary meeting notes, which recorded treatment that was planned, but not actually performed. There were 17 cases identified from EHIF that had claims for RT planning, but no procedures. The study demonstrated the ability to use insurance claims data to define variables of cancer treatment. This is particularly important, as the additional analysis showed inadequate completeness of RT data reported to ECR and using ECR data only could lead to underestimation of the use of RT.

The main limitations of this study are lack of data on comorbidities, performance status and molecular profile of tumours, which may seriously affect the choice of treatment and the administration of RT. We did not have any data on patient preferences, which may play a large role. However, we limited the analysis to surgically treated patients, as a proxy for overall health status or patient compliance, to account for factors that we were not able to measure. The focus was on sociodemographic factors, so examining the utilization of RT according to mode of surgery (BCS versus mastectomy) was beyond the scope of this paper. We did not account for type of surgery in regression modelling as social determinants impact the choice of primary treatment (BCS + RT or mastectomy) prior to surgery. A sensitivity analysis using BCS + RT as an outcome variable in early BC patients demonstrated similar associations for social factors, confirming the validity of our results.

During the whole study period of 2007–2018, half of the study population received RT within 12 months from diagnosis, which is comparable to data from the United States (51 % during 2009–2018) [40]. Overall RT utilization reached 58 % for 2016–2018, which is somewhat lower than the 63 % shown for England in 2013–2014 [41]. Stage-specific RT utilization in England was 70 %, 65 and 80 %, for stages I, II and III, respectively, compared to Estonian respective estimates of 68 %, 59 and 68 % during 2016–2018 (data not shown). The proportion of stage IV patients receiving RT was considerably lower in Estonia (11 % in 2016–2018, data not shown) compared to both United States and England.

Lower RT utilization in Estonia is in line with inferior availability of RT equipment – in 2012, the total number of MV units per million population in Estonia was 3.0, while it was 5.1 in England [14]. Although the total number of MV units reached six in 2016 (4.6 per million population), the estimated need is nine MV units according to Estonian Cancer Control Plan for 2021–2030 [42]. The difference between actual and optimal number of radiotherapy courses was estimated to be -1216 for 2013–2017 [42]. Nevertheless, our finding that RT utilization increased over time is consistent with increasing availability of RT equipment over the study period, and also with prior studies conducted in other countries including New Zealand and Canada [27, 29]. Besides lack of equipment, another possible reason for less frequent RT use in Estonia is higher prevalence of comorbidities. A previous study showed that among early BC patients, the proportion of women with no comorbidities was the lowest in Estonia among nine European countries [8].

Overall, patients aged 70 years and over had the lowest proportion of RT use, which was more than two times lower than for younger age groups. These findings are consistent with previous studies showing association between age at diagnosis and receipt of RT [27, 28, 30]. Less frequent RT use in women aged ≥ 70 years is in line with growing evidence over the time period under study suggesting no benefit for women in this age group in early disease [43, 44]. In multivariate analysis, RT utilization was the highest among women aged 50–59 and the difference from older and younger age groups was borderline significant. It has been reported previously that mortality from BC among patients aged 50–59 in Estonia has significantly declined since 2000, while it did not decrease among women aged ≥ 60 over the same time period [2]. Whereas these trends are consistent with screening activities, as women aged 50–59 have been the target age group for organized mammography screening since 2004 [20], the impact of RT in combination with other therapies can be considered as well. Women under 50 years of age were less likely to receive RT than women aged 50–59, which may be associated with higher proportion of genetically determined cancers in this age group and recommendations to use mastectomy rather than BCS and RT in cases with BC gene (BRCA) mutations [45]. Growing availability of immediate reconstruction may facilitate the choice of mastectomy in younger women.

In this study, no differences were observed by region of residence in multivariate analysis, even though overall receipt of RT was highest among women living in Southern Estonia and lowest among those living in Western Estonia. As living in rural areas and geographically remote areas have been shown to be one of the barriers to receipt of RT [26, 30–32], equal access to RT regardless of geographic factors in Estonia can be partly attributed to the opportunity to stay at the hospital for the duration of treatment, but also to urban life style of majority of Estonian residents. According to Statistics Estonia, approximately two-thirds of population in Estonia live in urban regions, and among people living in rural areas, those living in the suburbs of larger cities have urban lifestyles in which employment in agriculture has decreased [46].The finding of no association with place of residence is encouraging and suggests equal access to RT in Estonia regardless of geographic factors.

However, we observed significantly lower RT utilization among women with the lowest level of education, thus demonstrating an impact of social determinants on the use of RT. Educational level as an indicator of socioeconomic status has been shown to be associated with poorer health outcomes in Estonia, partly mediated by lower access to health care [17–19, 47]. Factors influencing receipt of optimal cancer treatment can be divided into three main categories: structural factors, factors affecting physician recommendation and factors affecting patient’s decision making [48]. Even though structural barriers such as lack of health insurance may affect timely presentation, all patients in Estonia who receive a cancer diagnosis obtain insurance coverage and consequently, access to standard treatment. Besides clinical factors, physicians’ recommendations may be influenced by their perception of patient’s ability to comply with treatments, while patient-related factors include socioeconomic status, access to transportation, ability to take time off from work, but also patients’ attitudes towards treatments and their beliefs [48]. Our finding that higher level of education was a considerable predictor of increased RT utilization is consistent with prior studies [28, 49] and may be related to both physician- and patient-related factors. Several studies have observed that patients with higher level of education were more likely to receive BCS in comparison to other types of surgeries [25, 50–52]. Patients with lower socioeconomic status level may struggle to cope with healthcare systems, have misperceptions about treatment benefits and may be more likely to have difficulties in overcoming adverse effects of treatment or psycho-social problems [48].

RT use was significantly associated with marital status, as divorced/widowed and single women received RT less often than married women. These findings are consistent with previous reports elsewhere [26, 53]. Furthermore, previous studies have shown marital status to be a predictor of cancer survival [54–58]. Longer survival of married individuals can be attributed to increased social support and improved economic status [59]. Studies have shown that unmarried women are more concerned about insufficient care after their treatment, and seeking help and transportation in comparison to married women and are thus more prone to refuse intense treatments and decline therapies such as axillary dissection and RT [60]. Such concerns can also have an impact on physician recommendations and physicians may be less likely to offer intense treatments to unmarried older women [57]. It might be beneficial for healthcare providers to identify unmarried women and provide comprehensive case managements to reduce health disparities. Studies have shown implementing nurse case management has improved receipt of RT among older BC patients, particularly those with poor social support [61].

## Conclusions

In conclusion, this study demonstrated the ability to use administrative databases as an additional source for identifying individual cancer treatment data if such data are not complete at a cancer registry. The study showed considerable increase in the use of RT in Estonia over the study period, which is in line with increases in available equipment. The lack of geographic variations suggests equal access to therapy for patients living in remote regions. However, unmarried women and those with lower educational level received less RT compared to their counterparts, revealing the impact of social factors on the use of health services. Further studies are needed to identify the exact mechanisms behind these findings, but some of the likely reasons are misperceptions about treatment benefits, loss of social support and economical disadvantages. The results have important implications for policymaking and evidence-based decisions. To minimise disparities in BC outcomes and avoid inequitable delivery of RT in Estonia, the healthcare and social system need a stronger focus on patient-centred care, offering patients psychosocial support, helping them cope with the disease and treatment effects and overcome any barriers to treatment.

## Data Availability

The datasets generated and analysed during this study are available from the corresponding author on reasonable request.

## Abbreviations

BC: Breast cancer
RT: RTRT
BCS: Breast conserving surgery
ECR: Estonian Cancer Registry
EHIF: Estonian Health Insurance Funds
ICD-O-3: 3^rd^Edition of International Classification of Disease for Oncology
ICD-10: International Statistical Classification of Diseases and Health Problems 10^th^ Revision
PRR: Prevalence rate ratio
CI: Confidence intervals
MV: Megavoltage
IMRT: Intensity modulated radiation therapy
IGRT: Image-guided radiation therapy
